# Circular RNA Expression Signatures Provide Promising Diagnostic and Therapeutic Biomarkers for Chronic Lymphocytic Leukemia

**DOI:** 10.3390/cancers15051554

**Published:** 2023-03-01

**Authors:** Ehsan Gharib, Parinaz Nasri Nasrabadi, Gilles A. Robichaud

**Affiliations:** 1 Département de Chimie et Biochimie, Université de Moncton, Moncton, NB E1A 3E9, Canada; 2 Atlantic Cancer Research Institute, Moncton, NB E1C 8X3, Canada

**Keywords:** cancer, chronic lymphocytic leukemia, circular RNA, diagnosis, prognosis, drug sensitivity prediction

## Abstract

**Simple Summary:**

This study aimed to evaluate the potential of circular RNA (circRNA) expression profiles for the early detection of chronic lymphocytic leukemia (CLL) using bioinformatic algorithms on verified CLL patient datasets. We analyzed and validated the diagnostic performance of circRNAs as potential biomarkers in different CLL sample sets, which reveal better prognostic value than existing clinical risk scales for the prediction of 5-year overall survival. The identification of specific circRNAs from our biomarker panel are also involved in cancer-related pathways, which possess druggable targets for therapeutic interests. Our findings therefore suggest that circRNA signatures represent significant biomarkers for the early detection and surveillance of CLL in addition to providing pharmacogenomic value to personalized medicine.

**Abstract:**

Chronic lymphocytic leukemia (CLL) is a known hematologic malignancy associated with a growing incidence and post-treatment relapse. Hence, finding a reliable diagnostic biomarker for CLL is crucial. Circular RNAs (circRNAs) represent a new class of RNA involved in many biological processes and diseases. This study aimed to define a circRNA-based panel for the early diagnosis of CLL. To this point, the list of the most deregulated circRNAs in CLL cell models was retrieved using bioinformatic algorithms and applied to the verified CLL patients’ online datasets as the training cohort (*n* = 100). The diagnostic performance of potential biomarkers represented in individual and discriminating panels, was then analyzed between CLL Binet stages and validated in individual sample sets I (*n* = 220) and II (*n* = 251). We also estimated the 5-year overall survival (OS), introduced the cancer-related signaling pathways regulated by the announced circRNAs, and provided a list of possible therapeutic compounds to control the CLL. These findings show that the detected circRNA biomarkers exhibit better predictive performance compared to current validated clinical risk scales, and are applicable for the early detection and treatment of CLL.

## 1. Introduction

Chronic lymphocytic leukemia (CLL) is one the most frequent hematologic cancers in the western world, and is responsible for one-third of all adult leukemia cases [1]. The National Institutes of Health’s (NIH) official estimation for CLL in the United States is more than 20,000 new cases and 4000 deaths for 2022 [2]. At the molecular level, CLL is characterized by the clonal expansion of neoplastic CD5^+^, CD19^+^, and CD23^+^ B-cells [3], which can elicit a wide range of heterogeneous clinical features from indolent to highly aggressive disease manifestations in CLL patients [4]. Suspected individuals are diagnosed with CLL if they express >5 × 10^9^/L mature lymphocytes co-expressing CD5, CD19, and CD23 [4], along with a potential mutation in the immunoglobulin heavy-chain variable-region (IGH_V_) [5] or in the ζ-associated protein 70 (ZAP-70) gene [6,7]. Validated clinical staging scales developed by Binet [8] and Rai [9] are the other prognosis assessment tools currently used for CLL diagnosis. However, due to the lack sufficient sensitivity and asymptomaticity in CLL patients, these techniques do not distinguish patients with early CLL from those in advanced stages of the disease [10], which is why the treatment process in patients with CLL is ineffective. Therefore, finding a fast and reliable diagnostic biomarker for CLL will bring significant benefits, and justifies further work in this area.

Circular RNAs (circRNAs) are a class of noncoding RNAs with a covalently continuous loop structure. They are mainly generated by back-splicing or lariat introns of two or more exons or introns [11]. Features such as abundancy [12], stability [13], and conservation [14] have created great consideration for circRNAs in various human disorders such as immune responses [15], cancer malignancies [16], cardiovascular events [17], neurological deficits [18], and metabolic diseases [19]. Mechanistically, circRNAs participate in signaling cascades through the sponging of small RNAs such as microRNAs [20]; the regulation of protein production by sequestering RNA-binding proteins (RBPs) [21], and even templating for protein synthesis through their open reading frames (ORFs) [22]. These properties support the potential analytical validity of circRNAs as better biomarkers over the canonical linear forms of RNAs in human diseases.

Over the past few years, clinical studies have highlighted the diagnostic performance of circRNAs in hematopoietic malignancies, including acute myeloid leukemia (AML) [23,24], acute lymphoblastic leukemia (ALL) [25,26], and chronic myeloid leukemia (CML) [27,28,29]. With regard to CLL, fewer attempts have been made to establish circRNA prognosis potency. So far, circRNAs circ-CBFB [30], mitochondrial genome-derived mc-COX2 [31], and plasma Circ-RPL15 [32] have shown good analytical value as biomarkers in CLL. Despite these efforts, a reliable biomarker capable of efficiently dissociate the early stages of CLL from fully developed cancer malignancies has yet to be identified. Considering the paucity of studies on dependable CLL biomarkers, we set out to find a signature gene circRNA profile capable of distinguishing early detection of CLL. Our data showed that the combination of circKAT6A, circLNPEP, circMDM2, and circMYH9 could successfully discern CLL cells from healthy B-lymphocytes and differentiate patients with early CLL from those in advanced stages.

## 2. Materials and Methods

### 2.1. Sample Size Estimation

The sample size for the study was calculated using the chi-square test with a significance level of α = 0.05 and a power of β = 0.2 [33]. Results from the training cohort revealed that the proportion of periodontitis was 0.31 in normal B-cells and 0.6 in CLL samples. The ratio of cancer cases to healthy controls was set at 1:1.9. Additionally, the sample size for the validation set I was increased by 10% due to the presence of a confounding variable (circRNA panel).

### 2.2. Omics Datasets Mining

The study population consisted of >600 CLL lymphocytes and B-cell RNA-sequencing data collected from the Gene Expression Omnibus (GEO) dataset series (GSE): GSE151159, GSE92626, GSE66117, GSE111014, GSE119103, GSE66121, GSE11154, GSE95352, GSE161711, GSE109085, GSE113386, GSE70830, GSE100026, GSE66228, GSE66167, GSE216288, GSE192685, GSE198454, GSE197811, GSE196741, GSE165087, GSE176141, GSE130385, GSE162427, GSE136634, GSE123777, and GSE111015, along with EMBL’s European Bioinformatics Institute (EBI) biostudies E-MTAB-12124 and -1176. Details regarding the number, sex, and staging of each set are shown in Table 1.

### 2.3. RNA-seq Processing and Normalization

First, the adaptor sequences of the Illumina paired-end reads were trimmed prior to mapping by Cutadapt [34] and then aligned to the UCSC human reference genome (GRCh37/hg19) using Bowtie2 [35]. The coding-protein reads were detected based on criteria defined by Coding-Noncoding-Index v2.0 [36], the Calculator-2 (CPC2) tool [37], InterPro [38], and PhyloCSF [39]. The Ensembl BioMart web-tool v101.0 (https://www.ensembl.org/Biomart, accessed on 5 October 2022) was used to sort the coding-reads based on the type (kinases, transcription factors, and other proteins).

To identify the circRNAs structures, 20mers from each end of the unmapped reads were extracted and aligned in an independent reversed mode (head-to-tail) to detect the back-spliced junction. Completed circular sequences were retrieved by extending the identified anchor alignments and flanking the GU/AG splice sites accordingly [40]. The circular reads were then annotated against the Circbase hg19 assembly reference file to identify the validated circRNAs [41]. CircRNA abundance was estimated by counting the total number of back-splices. The spliced reads per Billion Mapping (SRPBM) formula was used to assess the relative expression of reads [the total number of circular reads/(number of mapped reads × read length)].

As for the other genes, the expression level of reads was estimated by Cufflink software v2.2.0 and the Cuffdiff2 package 2.2.1 as reads per kilobase of transcript per million mapped reads (RPKM), indicating the total exon reads/mapped reads in millions × exon length in kb [42]. Differences were considered significant if the false discovery rate (FDR) and *q*-value (*p*-adjusted) < 0.05.

### 2.4. Enrichment Analysis

The interaction between CircRNAs and target miRNAs, kinases, transcription factors (TFs), and other proteins were analyzed based on StarBase v2.0 (http://starbase.sysu.edu.cn/, accessed on 22 October 2022), iCircRBP-DHN [43], and the BisoGenet v3.0 [44] algorithms, and were illustrated on Cytoscape platform v3.9.1 [45]. Network topology was visualized by CentiScaPe plugin v2.2 [46]. Annotations were deemed to be as significant if *p* < 0.05.

### 2.5. Drug Sensitivity Prediction

The response rate of the identified circRNA-related CLL signaling pathways to medicinal drugs and toxic chemicals was analyzed by the U.S. National Toxicology Program DrugMatrix (https://ntp.niehs.nih.gov/data/drugmatrix, accessed on 25 October 2022) and ToxicoDB (https://www.toxicodb.ca, accessed on 28 October 2022) databases. Estimations were assumed as statistically significant if *p* < 0.05.

### 2.6. Statistical Analysis

Statistical analyses were done by IBM SPSS Statistics software v26 (IBM, USA). Clinical variables among the studied cohorts were estimated using chi-square and unpaired unequal variance two-tailed Student’s t-tests. The Wilcoxon rank sum test was employed to assess the median differences of the genes. Spearman’s rank correlation coefficient analyzed the correlation between the differentially expressed genes and the clinicopathologic features of patients. Receiver operating characteristic (ROC) curves were plotted to determine the performance of circRNAs in diagnosing CLL. The calibration and discriminative abilities of the panel were calculated with the Hosmer–Lemeshow test and Harrell’s concordance index, respectively. The Kaplan–Meier survival curve was drawn to estimate patients’ 5-year overall survival (OS). We used univariate/multivariate Cox proportional hazards regression to detect the factors that independently affect the patients’ OS. All data are represented as the mean ± S.D. (Standard deviation) and taken as significant if *p* < 0.05 (*).

## 3. Results

### 3.1. The Expression Pattern of 52 circRNAs Was Significantly Different in CLL Lymphocytes

The initial population study comprised 635 CLL patients’ data profiles and 39 healthy B-cells, including 349 men and 325 women. However, due to insufficient clinical data, 64 CLL samples were removed from the final study. The remaining cohort was then divided into three independent sets, including a training set (100 CLLs) and two validation sets containing 220 (set I) and 251 (set II) CLL samples, respectively. The healthy B-cell data profiles were considered normal controls for each set. Additional details are shown in Table 1.

**Figure 1:**
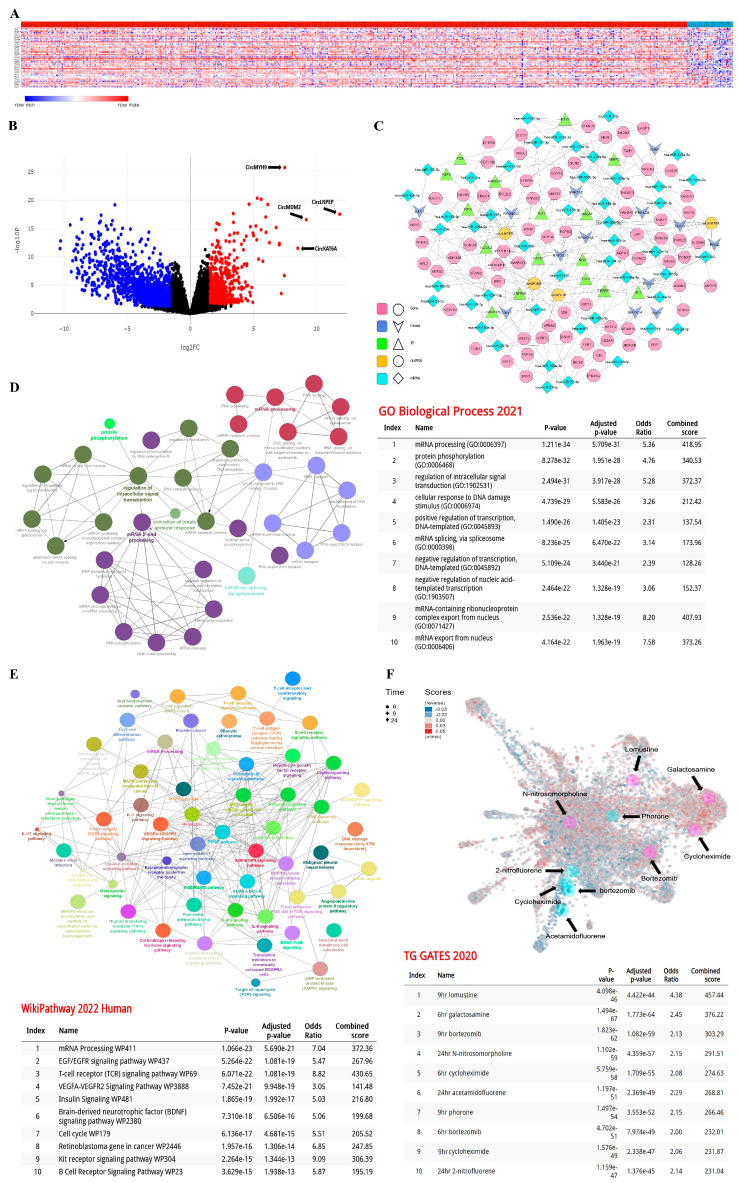
Bioinformatics analysis of circRNAs in CLL samples. (**A**) Heatmap demonstration of 52 differentially expressed circRNAs between CLL lymphocytes (red bar, right) and normal B-cells (blue bar, left). (**B**) Volcano plotting of deregulated circRNAs in CLL samples. (**C**) Interaction analysis between candidate circRNAs and other signaling mediators. (**D**) Gene ontology (GO) biological process and (**E**) WikiPathways enrichment analysis of gene networks. (**F**) Drug sensitivity prediction of gene networks in CLL.

The identification of circRNAs in processed CLL RNA profiles was made in accordance with the reference file provided by the Circbase database. Using a Log2 ≥ 2 as criteria, 52 circRNAs were labeled as differentially expressed in CLL samples versus healthy B-lymphocytes, including circC5orf25, circCHPT1, circPDE4B, circDENND2D, circRHOC, circPSMB4, circSEMA4A, circTP53BP2, circEIF4G2, circSRPR, circRAPGEF3, circPFDN5, circNCKAP1L, circGCN1L1, circSFSWAP, circMETTL17, circCCNDBP1, circGNB5, circVPS4A, circCYB5B, circCDK10, circNLRP1, circTP53I13, circSTARD3, circTCF3, circPRKCSH, circKLF1, circSAMD1, circNDUFA13, circNUCB1, circDNTTIP1, circTH1L, circPRIC285, circZMAT5, circMYH9, circLNPEP, circCD164, circCHMP7, circZNF395, circVCP, circMDM2, circKTN1, circVASH1, circKAT8, circTCF4, circPRDM2, circRALGPS2, circENTPD6, circCAB39, circHPS3, circKAT6A, and circGBA2 (Figure 1A,B). The interactions between these circRNAs and corresponding genes, kinase enzymes, TFs, and miRNAs were then estimated and visualized by bioinformatic algorithms (Figure 1C). According to the data, four circular RNAs: circKAT6A, circLNPEP, circMDM2, and circMYH9, play a crucial role in CLL signaling cascades. To better understand their impact on cancer progression, the obtained genes list was subsequently submitted for pathway analysis. Gene ontology (GO) enrichment assessment of these genes showed that they are actively involved in biological process pathways (Figure 1D) such as mRNA processing (GO:0006397), protein phosphorylation (GO:0006468), the regulation of intracellular signal transduction (GO:1902531), and cellular response to DNA damage stimulus (GO:0006974). Meanwhile, another enrichment tool, WikiPathways (https://www.wikipathways.org, 10 December 2022 Release) highlighted (Figure 1E) the gene network impact in the mRNA processing pathway (WP411), EGF/EGFR signaling pathway (WP437), T-cell receptor (TCR) signaling pathway (WP69), and the VEGFA-VEGFR2 signaling pathway (WP3888). Per these findings, we made a list of medicinal drugs and toxic chemicals with a potency to suppress the identified signaling pathways (Figure 1F). Our model showed that compounds such as lomustine, galactosamine, bortezomib, N-nitrosomorpholine, and cycloheximide have an inhibitory impact on CLL progression and can be considered for therapeutic interventions. Details regarding the expression comparisons, pathways annotations, and drug sensitivity prediction are provided in Supplementary Materials S1–S4, respectively.

### 3.2. Establishment of the circRNA-Biomarker Panel

Next, we tested the diagnostic performance of candidate circRNAs as individual biomarkers for dissociating CLL lymphocytes from healthy B-cells in the training set. According to the results, four circRNAs, including circKAT6A, circLNPEP, circMDM2, and circMYH9, had a good predictor value (AUC > 0.7) among the examined RNAs (Table 2). These circRNAs were thus considered for subsequent experiments.

To develop the diagnostic circRNA-biomarker panel, we combined the individual circRNA biomarkers and determined a new biomarker risk score (BRS) with the regression model as previously reported [47,48,49]. To this point, the expression level of each circRNA was recalculated as a log2-transformed variable to lower the variation between each biomarker and the logistic regression coefficient generation. The BRS of each sample was then determined as the sum of each circRNA risk score yielded by multiplying the level of a circRNA by its corresponding coefficient (Risk score = ∑ logistic regression coefficient of circRNA × expression level of circRNA). The median BRS was then used as the cutoff point for dividing the high-risk samples from the low-risk group.

**Figure 2:**
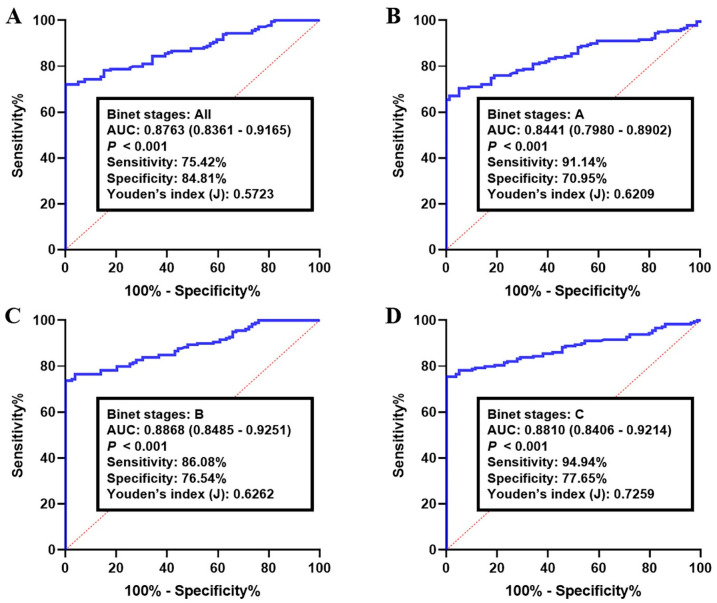
Receiver operating characteristics (ROC) curve analysis of the log *it* model with the circKAT6A/circLNPEP/circMDM2/circMYH9 panel in the training set. The study group consisted of 100 CLLs and healthy B-lymphocytes. Using the optimal cutoff value of 0.7262, the diagnostic performance of the circRNA panel for discriminating CLLs with (**A**) all Binet stages, (**B**) Binet stage A, (**C**) Binet stage B, and (**D**) Binet stage C from healthy samples were examined. Log *it* (p) of the model was 0.7262 + 0.7690 × CircKAT6A + 0.1786 × CircLNPEP − 0.2246 × CircMDM2 + 0.9938 × CircMYH9.

The most optimal combination of the candidate circRNA biomarkers was identified by establishing a stepwise logistic regression coefficients model between CLLs and healthy B-lymphocytes in the training set. We then constructed the ROC curve using the log *it* model of 0.7262 + 0.7690 × CircKAT6A + 0.1786 × CircLNPEP − 0.2246 × CircMDM2 + 0.9938 × CircMYH9 (Table 3). Considering 0.7262 as the optimal cutoff point, the training set CLL samples were divided into a high-risk and a low-risk score group. The obtained results indicated a higher diagnostic accuracy of the combination of these circRNAs as a panel compared to their performance as individual biomarkers, along with a suitable adjustment of the model to the data (Hosmer–Lemeshow test, *p* = 0.13) (Figure 2). For all Binet stages (A–C), the area under the roc curve (AUC) of the circRNA-biomarker panel was 0.8763 (95% CI: 0.8361–0.9165, sensitivity: 75.42% and specificity: 84.81%, Figure 2A). Meanwhile, the analysis of the Binet stage A CLL samples showed an AUC of 0.8441 (95% CI: 0.7980–0.8902, sensitivity: 91.14% and specificity: 70.95%, Figure 2B). As for Binet stage B, the obtained AUC was 0.8868 (95% CI: 0.8485–0.9251), with a sensitivity of 86.08% and a specificity of 76.54% (*p* < 0.001, Figure 2C). Accordingly, the ROC curve analysis of CLL samples with Binet stage C yielded an AUC of 0.8810 (95% CI: 0.8406–0.9214) followed by a 94.94% sensitivity and 77.65% specificity (*p* < 0.001, Figure 2D).

### 3.3. Validation of the circRNA-Biomarker Panel

**Figure 3:**
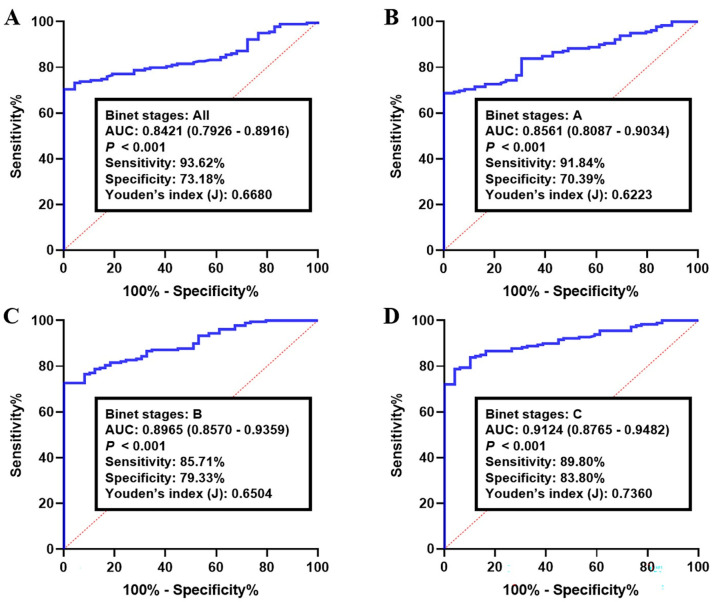
Receiver operating characteristics (ROC) curve analysis of the log *it* model with the circKAT6A/circLNPEP/circMDM2/circMYH9 panel in the validation set I. The study group consisted of 220 CLLs and healthy B-lymphocytes. Using the optimal cutoff value of 0.7262, the diagnostic performance of the circRNA panel for discriminating CLLs with (**A**) all Binet stages, (**B**) Binet stage A, (**C**) Binet stage B, and (**D**) Binet stage C from healthy samples were examined. Log *it* (p) of the model was 0.7262 + 0.7690 × CircKAT6A + 0.1786 × CircLNPEP − 0.2246 × CircMDM2 + 0.9938 × CircMYH9.

The diagnostic accuracy of the circRNA-biomarker panel was then examined in the validation set I and II, consisting of 220 and 251 CLL samples, respectively. The corresponding AUC for all Binet stages (A–C stages) in validation set I was 0.8421 (95% CI: 0.7926–0.8916; sensitivity: 93.62% and specificity: 73.18%, Figure 3A). The analyzing of CLL lymphocytes with Binet stage A resulted in an AUC of 0.8561 (95% CI: 0.8087–0.9034) with a sensitivity and specificity of 91.84% and 70.39%, respectively (*p* < 0.001, Figure 3B). The AUC of Binet stage B-CLLs was 0.8965 (95% CI: 0.8570–0.9359; sensitivity: 85.71% and specificity: 79.33%, Figure 3C). As for the CLLs with Binet stage C, the median AUC was higher than the other conditions (0.9124, 95% CI: 0.8765–0.9482), with a sensitivity of 89.80% and a specificity of 83.80% (*p* < 0.001, Figure 3D).

**Figure 4:**
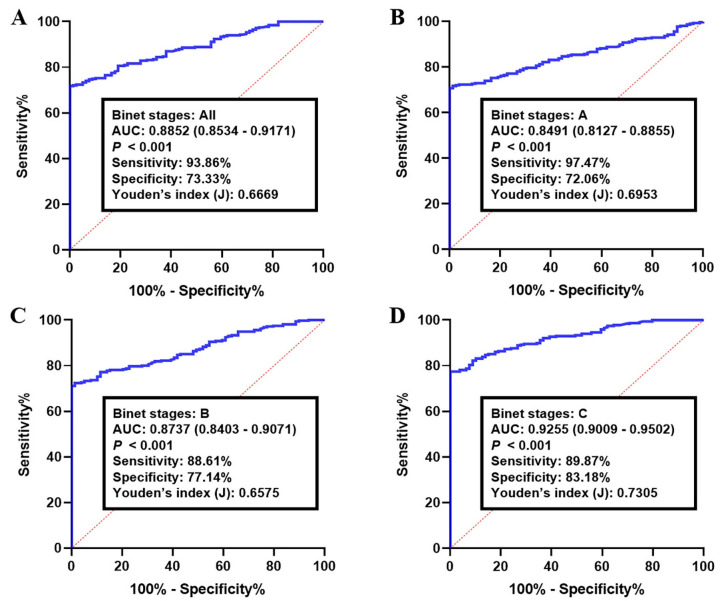
Receiver operating characteristics (ROC) curve analysis of the log *it* model with the circKAT6A/circLNPEP/circMDM2/circMYH9 panel in the validation set II. The study group consisted of 251 CLLs and healthy B-lymphocytes. Using the optimal cutoff value as 0.7262, the diagnostic performance of circRNA panel for discriminating CLLs with (**A**) all Binet stages, (**B**) Binet stage A, (**C**) Binet stage B, and (**D**) Binet stage C from healthy samples were examined. Log *it* (p) of the model was 0.7262 + 0.7690 × CircKAT6A + 0.1786 × CircLNPEP − 0.2246 × CircMDM2 + 0.9938 × CircMYH9.

ROC curve analysis of the proposed circRNA-biomarker panel in validation set II indicated a similar diagnostic efficacy in a larger sample population. The corresponding AUC for all CLLs (Binet A–C stages) was 0.8852 (95% CI: 0.8534–0.9171), with a sensitivity of 93.86% and a specificity of 73.33% (*p* < 0.001, Figure 4A). Accordingly, the diagnostic accuracy of the panel in Binet stage A samples was 0.8491 (95% CI: 0.8127–0.8855; sensitivity: 97.47% and specificity: 72.06%, Figure 4B), while CLLs with Binet stage B showed an AUC of 0.8737 (95% CI: 0.8403–0.9071; sensitivity: 88.61% and specificity: 77.14%, Figure 4C). Meanwhile, the analyzing of CLL data profiles with Binet stage C resulted in an overall AUC of 0.9255 (95% CI: 0.9009–0.9502) along with a sensitivity of 89.87% and a specificity of 83.18% (*p* < 0.001, Figure 4D).

### 3.4. Prognostic Performance of circRNA-Biomarker Panel in CLL

A Cox’s proportional hazards model determined the prognostic value of the circRNA-biomarker panel in CLL lymphocytes. To this point, the clinical features of patients, including gender, age, and Binet stage, were added to the model for a better conclusion (Table 4). According to the univariate data of clinical variables, Binet staging had good prognostic efficacy in validation sets I (HR: 2.829, *p* < 0.01) and II (HR: 3.492, *p* < 0.01), but not in the training set (HR: 2.119, *p* = 0.071). Other parameters did not show any statistically significant prognostic impact in the studied groups.

We evaluated the prognostic performance of the circRNA-biomarker panel as unadjusted and in combination with the Binet stage parameter by multivariate analysis (Table 5). The univariate Cox regression model reported that the combination of circKAT6A, circLNPEP, circMDM2, and circMYH9 deregulations had a significant impact on OS in the training set (HR (95% CI): 6.083 (5.152–8.023), *p* < 0.001), validation set I (HR: 7. 517 (6.117–8.886), *p* <0.001), and validation set II (HR: 8.294 (7.503–9.167), *p* < 0.001). Multivariate Cox regression analyses adjusting for the circRNA-biomarker panel also resulted in a good prognostic value of 4.193 in the training set (95% CI: 3.122–5.243, *p* < 0.001), along with HR values of 5.432 (95% CI: 4.716–6.915) and 6.992 (5.9365–8.049) in validation set I and II, respectively (*p* < 0.001, Table 5).

**Figure 5:**
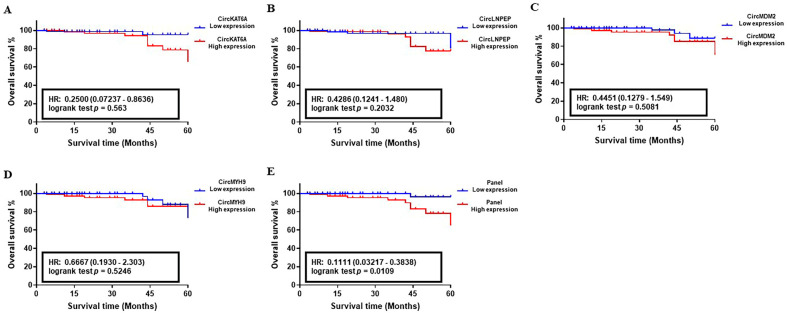
Correlation between the abnormal expression of candidate circRNAs and overall survival (OS) of CLL in the training set (*n* = 100). (**A**–**D**) Survival impact of CircKAT6A, CircLNPEP, CircMDM2, and CircMYH9 as individual biomarkers, and (**E**) in the form of a panel in CLL. Kaplan-Meier analysis indicated a reverse correlation between the high level of combined circRNAs and the poor survival of CLL patients in training set I.

**Figure 6:**
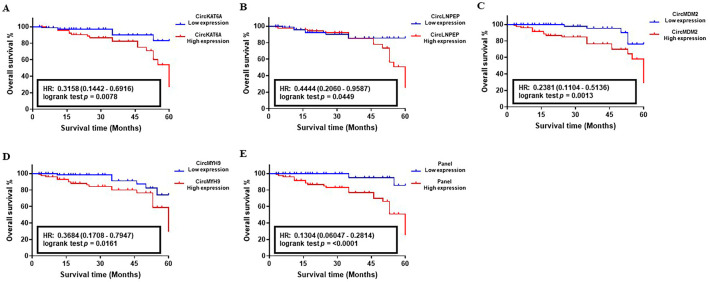
Correlation between the abnormal expression of candidate circRNAs and overall survival (OS) of CLL in validation set I (*n* = 220). (**A**–**D**) Survival impact of CircKAT6A, CircLNPEP, CircMDM2, and CircMYH9 as individual biomarkers, and (**E**) in the form of a panel in CLL. A Kaplan-Meier analysis indicated a reverse correlation between the high level of circRNAs individually and combined, and the worse survival of CLL patients in validation set I.

**Figure 7:**
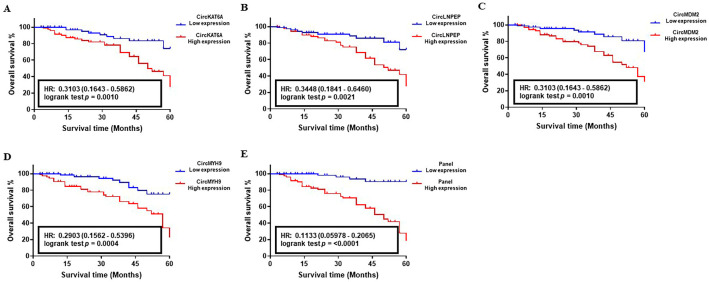
Correlation between the abnormal expression of candidate circRNAs and overall survival (OS) of CLL in validation set II (*n* = 251). (**A**–**D**) Survival impact of CircKAT6A, CircLNPEP, CircMDM2, and CircMYH9 as individual biomarkers, and (**E**) in the form of a panel in CLL. A Kaplan-Meier analysis indicated a reverse correlation between the high level of circRNAs individually and combined, and the worse survival of CLL patients in the validation set II.

The correlation between the abnormal level of circKAT6A, circLNPEP, circMDM2, and circMYH9 RNAs and 5-year OS of CLL patients was determined using the Kaplan–Meier survival analysis. The median follow-up of patients in the studied cohorts was 58 months with an average of 83.8% (Training set), 85.1% (Validation set I), and 88.3% (Validation set II). The analysis of the target circRNAs in the training set showed an HR value of 0.25 (CircKAT6A, *p* = 0.563), 0.4286 (CircLNPEP, *p* = 0.2032), 0.4451 (CircMDM2, *p* = 0.5081), and 0.6667 (CircMYH9, *p* = 0.5246), indicating their poor impact on patients’ survival as individual biomarkers (Figure 5A–D). On the contrary, the OS analysis of candidate circRNAs in larger population cohorts proved their correlation with worse clinical outcomes of the CLL patients in such a way that abnormal levels of circKAT6A, circLNPEP, circMDM2, and circMYH9 resulted in an HR of 0.3158, 0.4444, 0.2381, and 0.3684, respectively, in validation set I (*p* < 0.05, Figure 6A–D). Similar observations were obtained in the validation set II, and patients with a high level of circKAT6A (HR: 0.3103), circLNPEP (HR: 0.3448), circMDM2 (HR: 0.3103), and circMYH9 (HR: 0.2903) had a worse OS than those with a low-expression group (*p* < 0.01, Figure 7A–D). On the other hand, the combination of these deregulations as a panel had a statistically significant impact on CLL patients’ OS and yielded an overall HR of 0.1111 (*p* < 0.05, training set, Figure 5E), 0.1304 (*p* < 0.001, validation set I, Figure 6E), and 0.1133 (*p* < 0.001, validation set II, Figure 7E). These data showed that the combination of circKAT6A, circLNPEP, circMDM2, and circMYH9 as a panel has higher prognostic accuracy than the other clinical parameters used in this study.

## 4. Discussion

Chronic lymphocytic leukemia (CLL) is the most common form of leukemia, and accounts for approximately 30% of all adult leukemias [50]. Pathologically, CLL is a B-cell malignancy that is characterized by the relentless accumulation of slow proliferating (or quiescent), immunologically dysfunctional, mature B-lymphocytes that fail to undergo apoptosis (reviewed in [51]). Although the first clinical description of CLL was published over 150 years ago, it is still considered an “enigma” of modern hematology, as complete remission for this disease remains elusive today [52]. The course of disease progression can also vary significantly amongst individuals. For instance, some CLL patients experience indolent disease that progresses very slowly over several years and does not require immediate treatment. On the other hand, others characterized with cytogenetic biomarkers associated with CLL manifest more aggressive progression combined with drug therapy resistance.

CLL malignancy is clinically determined by prognostic factors determined by genotyping analyses. The latter genetic alterations are also used to determine the therapeutic plan of CLL patients, such as the FCR regimen (fludarabine, cyclophosphamide, rituximab) [53]. Despite the characterization of these CLL biomarkers, genotyping approaches have proven to be complex (ex: IGHv status), and CLL is still not associated with a specific cytogenetic defect [54]. Consequently, CLL remains uncurable due to disease recidivism caused by chemoresistance. The study of predictive biomarkers for CLL is thus pivotal for early diagnosis, monitoring, and to define the most appropriate therapeutic approach to improve disease outcome [51,53].

The purpose of this study was to determine circRNA expression profiles in CLL patients to establish specific signatures associated with CLL disease. In addition, we wanted to determine the potential role of characterized circRNAs in the onset of disease, as well as their relationship with other prognostic markers and patient clinical status. CircRNAs represent a new class of non-coding RNAs, which are formed through the back-splicing of linear mRNAs, which results in the binding of both extremities to form a continuous circular RNA molecule [11]. CircRNAs have thus garnered interest as biomarker candidates given that they are highly resistant to RNase activity (due to the lack of 5′ and 3′ ends), and they are often expressed in tissue- and developmental stage-specific manners [55,56]. Studies have also shown that the aberrant expression of circRNAs is associated with cancer processes leading to disease progression [57,58,59]. More importantly, circRNA signaling pathways and function have been linked to tumor drug chemoresistance, including leukemia [60,61,62]. In this study, we tested 571 online CLL datasets and found 52 circRNAs where their expression is significantly modulated in cancer lymphocytes when compared to normal B-cells. CircRNA expression levels are usually tightly regulated as they control a broad network of genes, miRNAs, kinases, and TFs through direct or indirect molecular interactions. By minimizing the obtained network, we revealed four circRNAs with high betweenness degrees that significantly impacted cancer-related signaling cascades. A more in-depth analysis revealed that their diagnostic and prognostic efficacies, individually and in the form of a panel, were higher than standard clinical parameters, and might be considered for the early detection of CLL.

Amongst the relevant differentially expressed circRNAs in CLLs, four circRNAs, including circKAT6A, circLNPEP, circMDM2, and circMYH9, have notably received prior focus for their regulation of cancer pathways. For example, circKAT6A is a circRNA derived from the Lysine acetyltransferase-6A (*KAT6A*) gene coding sequence. The *KAT6A* gene itself encodes an MYST-type histone acetyltransferase (HAT) enzyme, which is essential for the maintenance of hematopoietic stem cells [63], controlling cell cycle progression [64], and inducing cell senescence [65]. Subsequently, the oncogenic role of KAT6A has been highlighted in leukemia [66,67,68], breast cancer [69], and glioma [70]. As for the circKAT6A, this isoform was recently detected as one of the most abundantly expressed circRNAs in the oral squamous cell carcinoma (OSCC) cells, and is involved in survival responses [71]. However, the exact involvement of circKAT6A in cancer development is still not well defined. Accordingly, our network model showed that circKAT6A could control the platelet-derived growth factor (PDGF) signaling pathway, cholecystokinin (CCKR) signaling, and B-cell activation in CLL. These findings indicated that circKAT6A upregulation in CLL lymphocytes directly associates with cancer initiation and development.

Similar to circKAT6A, circLNPEP was also associated with CLL progression. At the protein level, the Leucyl and cystinyl aminopeptidase (*LNPEP*) gene encodes a zinc-dependent aminopeptidase that facilitates the antigen transportation and processing [72] involved in inflammatory responses in cardiovascular complications and diabetes mellitus [73,74]. In cancer, LNPEP deregulation has been shown to contribute to immune infiltration of ovarian cancer [75]. In the circular RNA form, LNPEP was reported to enhance Ras-related protein Rab-9A expression levels by sponging miR-532–3p under hypoxia and promoting invasiveness in hepatocellular carcinoma cells [76]. The role of circLNPEP in ovarian cancer development was recently reported by Wang et al. (2021), where the authors claim that circLNPEP sponging of miR-876-3p diminished its inhibitory impact on WNT5A, which results in cancer cell growth and survival [77]. In line with these findings, our data demonstrated that circLNPEP could sponge the tumor suppressor miRNAs miR-15a-5p, miR-19a-3p, miR-135a-5p, and miR-138-5p, which are involved in specific activation pathways supporting protein acetylation, RNA polymerase II-dependent transcription, and the regulation of intracellular signal transduction in CLL cells, respectively. Our findings thus introduce circLNPEP as a key player in CLL growth and progression.

The next circRNA in our network with a high impact on CLL was circMDM2, a circular RNA from the mouse double minute 2 homolog (MDM2) locus [78]. The *MDM2* gene encodes a cellular phosphoprotein that acts as a negative regulator of the p53 tumor suppressor, and is upregulated in many human cancer malignancies [79]. As with its protein counterpart, the circular form of MDM2 is involved in cancer development by decreasing p53 and p21 levels [79]. More interestingly, it has been shown that elevated levels of circMDM2 in response to DNA damage in colorectal cancer (CRC) cell lines lowered MDM2 protein production, suggesting that this circular isoform is a derivative of the original pre-mRNA [79]. Nevertheless, the oncogenic impact of circMDM2 is not limited to p53 inhibition. A study by Zhang et al. (2020) on OSCC showed that the circular isoform of MDM2 could provoke proliferation and glycolysis in cancer cells through the sponging of the miR-532-3p, thus impacting downstream hexokinase 2 (HK2) levels [80]. The authors also showed that patients with a higher level of circMDM2 had poorer survival than those expressing low circMDM2 levels. Following these observations, the current investigation has identified a wide range of signaling mediators, including Nuclear factor I X (NFIX), Syntrophin Beta-2 (SNTB2), and RAF1, which interact with the circMDM2 directly or indirectly through miR-137, the miR-193 family, or miR-7, respectively. These interactions enable circMDM2 to actively manipulate the cell cycle, apoptosis, and PDGF signaling pathways in CLL cells, and to subsequently boost cancer development.

The final member of our list is circMYH9, which derives from an intron of the Myosin Heavy Chain-9 (MYH9) transcripts sequence [81]. The *MYH9* gene is a well-known oncogene that is directly associated with progression, invasiveness, and drug resistance in many human cancers [82]. Accordingly, the MYH9 circular product has recently been reported to play a similar role in cancer cells’ fate by increasing the mRNA stability of Karyopherin α2 (KPNA2), another known oncogene in hepatocellular carcinoma [83]. CircMYH9 also promotes CRC development by degrading p53 pre-mRNA and altering cell metabolism and redox homeostasis [81]. More interestingly, the enhanced level of circMYH9 in CRC cells has been shown to increase hepatoma-derived growth factor (HDGF) mRNA stability by sponging the tumor suppressing miRNA miR-761, leading to increased cancer cells survival against the anticancer compound Baicalin [84]. In corroboration, our analysis expands on the underlining gene network of circMDM2 to reveal a possible impact on angiogenesis, B-cell activation, and the EGF receptor signaling pathway through the interaction with NCK2, Casitas B-lineage lymphoma (CBL), and the Signal transducing adaptor molecule-2 (STAM2) oncogene. These findings demonstrate a direct correlation between the aberrant expression of circMYH9 and CLL cancer progression.

A common challenge following the detection of the deregulated signaling pathways in cancer cells such as CLL is how to utilize these data for the development of strategic therapeutic and/or diagnostic interventions. To shed insight into the potential application of our list, we analyzed the yielded gene networks with different algorithms and made a list of potential drugs, such as lomustine, galactosamine, and bortezomib, that could target essential pathways identified in our gene networks. In fact, a comprehensive literature survey showed that these drugs had previously been applied for hematopoietic malignancies [85,86]. For example, Pigneux et al. (2010) used the alkylating agent lomustine as a therapeutic intervention for elderly patients with de novo AML. The authors reported that combining lomustine with idarubicin and cytarabine as standard therapy improved patients’ complete remission (CR) and survival [85]. Similarly, a regimen consisting of lomustine, mitoxantrone, and vinblastine was shown to be effective in patients with Hodgkin’s disease without confronting the adverse side effects and toxicity associated to compounds like bleomycin [86]. Despite its potency, lomustine has not been tested in human CLL subjects. Still, promising evidence has been published regarding the efficacy of lomustine combined with vincristine, procarbazine, and prednisolone in animal-harbored T-CLL [87,88,89], suggesting lomustine as a reliable chemotherapeutic agent for the control of hematological cancer growth. In addition, our drug sensitivity model based on circRNA interaction networks predicted over 900 medicinal drugs and toxic chemicals that could be used for therapeutic interventions in CLL. Alternatively, our data could also support the pharmacogenetic evaluation of patients to develop a more personalized therapeutic approach and benefit the outcome of CLL patients.

Given the fact that CLL diagnosis uses a combination of criteria, the disease may encompass multiple related conditions [52,90]. This heterogeneity is reflected by the variability in CLL biology in terms of the location of the disease, potential cell of origin, CLL-related symptoms, rate of progression, response to treatments, and overall survival time from diagnosis [91]. The clinical presentation of CLL biomarkers and their use are therefore our most effective and promising tools for disease outcome [92,93]. A limitation of current biomarker tests is that they produce continuous rather than binary results, meaning that marker levels do not correspond to a precise or predicted clinical outcome for the patient. For example, while ZAP-70 is commonly used as a biomarker for CLL diagnosis [7], the expression levels of this kinase can range from 0 to 100% in CLL cells from an individual patient. Therefore, elevated ZAP-70 levels would at best only partially correlate with CLL progression risk [94]. Another challenge in CLL biomarker profiling is that biosignatures and the reliability of their predictive value are constantly influenced by various biological characteristics such as microenvironment interactions, bioenergetic constraints, risk factors (behavioral, demographic, and environmental), and levels of other biomarkers [94]. Therefore, features like stability, abundance, and tissue specificity better qualify circRNAs as worthy biomarkers for cancer prognosis [23,24,25,26,27,28,29].

Cancer bioinformatics play a crucial role in identifying and validating biomarkers related to early diagnosis, disease progression, therapy response, and quality of life. It involves the study of biomarkers such as genes, proteins, peptides, and chemical and physical variables in cancer, from single to multiple markers, from expression to function, and from network to dynamic network. Network biomarkers (which are based on protein-protein interactions) are being investigated by integrating protein annotations, interactions, and signaling pathways. Dynamic network biomarkers (which can be monitored and evaluated at various stages of disease development) are expected to be linked to clinical informatics, including patient symptoms, history, therapies, clinical exams, biochemistry, imaging, pathology, and other measurements [95].

Systems clinical medicine is seen as a new approach to developing cancer biomarkers. This approach integrates systems biology, clinical phenotypes, high-throughput technologies, bioinformatics, and computational science to improve disease diagnosis, treatment, and prognosis. For a cancer biomarker to be effective, it should possess properties such as a network, dynamics, interaction, and specificity to disease. Understanding the relationship between clinical informatics and bioinformatics is the key to developing new diagnostics and treatments. This approach has been applied to other diseases, such as acute renal transplant rejection and lung disease [96,97]. In essence, human samples from clinical studies are collected and analyzed with a complete profile of clinical informatics. Gene and/or protein profiles are then analyzed, and dynamic networks and interactions between genes and/or proteins are determined through bioinformatics and systems biology.

The correlation between disease-specific and dynamic networks of genes/proteins with clinical phenotypes is achieved through computational analysis in order to validate and optimize disease-specific biomarkers. However, various challenges still persist in the implementation of systems clinical medicine, including the optimal translation of clinical descriptions into clinical informatics, the bioinformatics analysis that takes into consideration the disease severity, duration, location, and sensitivity to therapies, as well as the integration of clinical and high-throughput data for accurate conclusions. Additionally, determining the variations and significance between molecular networks, between molecular networks and clinical phenotypes, and between gene/protein interactions and expressions is also a challenge. Therefore, incorporating protein network and interaction data can enhance the interpretation of gene signatures, as demonstrated before by the efficacy of R-weighted Recursive Feature Elimination and average pathway expression in stratifying breast cancer patients [98].

Some limitations should be acknowledged when interpreting the results of this study. The main limitation arises from the lack of a mutual clinical parameter list. Although all of the samples studied had been classified based on their Binet stages, CLL diagnosis benefits from new prognostic markers such as IGHv variabilty or del(17p)/TP53 mutational status in CLL patients. For instance, an unmutated status of IGHV is determined when the immunoglobulin heavy-chain sequence of the CLL has less than a 2% difference in base pair sequences compared to a reference germline sequence [99]. This status is linked to a poorer prognosis and is present in approximately 40% of CLL cases upon diagnosis [54]. Conversely, mutated IGHv occurs when the CLL sequence has a difference of 2% or greater from the germline heavy-chain sequence, and is associated with a much better clinical prognosis [54,99]. The difference in overall survival between these two categories is significant. Due to its apparent association with the median survival of CLL patients, IGHv is now being used to assist clinicians with treatment decisions and to identify individuals who may benefit from modern therapies, such as ibrutinib, a BTK inhibitor [100]. Therefore, it would be important and interesting if we could determine the correlation between our circRNA biomarkers and these new prognostic markers to better understand the mechanisms involved in the pathogenetic process of CLL.

## 5. Conclusions

Altogether, our study assessed the diagnostic and prognostic value of circRNAs in CLL samples, and found that a panel of circKAT6A, circLNPEP, circMDM2, and circMYH9 has the potency to differentiate the B-CLLs from healthy lymphocytes and could potentially discriminate between patients with early benign cases from those with advanced stages of disease. Specifically, we showed that CLL cases with an increased level of circKAT6A, circLNPEP, circMDM2, and circMYH9 reveal lower overall survival rates. To our knowledge, this study is also the first to report the prognostic impact of circKAT6A and circMYH9 in human cancers. Further studies are thus warranted to elucidate the role of circKAT6A, circLNPEP, circMDM2, and circMYH9 in CLL disease to further establish their reliability as prominent biomarkers for prognostics, therapeutic planning, and the monitoring of disease progression.

## Figures and Tables

**Table 1:** Clinical features of the studied population.

Variable	Training Set (%)	Validation Set I (%)	Validation Set II (%)	*p* Value
Healthy count (%)				
Sex				-
Male	12 (30.77)	12 (30.77)	12 (30.77)	
Female	17 (69.23)	17 (69.23)	17 (69.23)	
Age (year)				-
Mean + SD	50 ± 9	50 ± 9	50 ± 9	
Chronic Lymphocytic Leukemia count (%)				
Sex				0.416
Male	31 (31.00)	121 (55.00)	163 (64.94)	
Female	69 (69.00)	99 (45.00)	88 (35.06)	
Age (year)				0.762
Mean + SD	69 ± 3	71 ± 2	70 ± 2	
Binet stage				0.109
A	23 (23.00)	33 (15.00)	34 (13.55)	
B	56 (56.00)	67 (30.45)	133 (52.99)	
C	21 (21.00)	120 (54.55)	84 (33.46)	

**Table 2:** Diagnostic performance of circRNAs in CLL.

RNA	Sensitivity	Specificity	Youden’s Index J	AUC	*p* Value	95% CI
CircKAT6A	66.67	76.32	0.4299	0.7828	<0.0001	0.6993–0.8664
CircLNPEP	62.67	71.05	0.3372	0.7498	<0.0001	0.6587–0.8410
CircMDM2	65.33	78.95	0.4428	0.8025	<0.0001	0.7207–0.8842
CircMYH9	58.67	73.68	0.3235	0.7504	<0.0001	0.6600–0.8407

AUC: Area under the ROC Curve, CI: Confidence Intervals, CircRNA: Circular RNA, and CLL: Chronic Lymphocytic Leukemia.

**Table 3:** Logistic regression of circRNAs in the training set.

Variable	Coefficient	Std. Error	Odds Ratio	95% CI	*p* Value
CircKAT6A	0.7690	0.1042	0.9260	0.1089–2.252	<0.0001
CircLNPEP	0.1786	0.0781	0.8365	0.09177–1.043	<0.0001
CircMDM2	0.2246	0.1213	1.445	1.028–3.811	<0.0001
CircMYH9	0.9938	0.1745	2.701	0.3728–3.241	<0.0001
Constant	0.7262				

CI: Confidence Intervals, CircRNA: Circular RNA, and Std. Error: Standard Error.

**Table 4:** Univariate Cox regression analysis of overall survival of the clinical variables in CLL patients.

		Training Set (*n* = 100)		Validation Set I (*n* = 220)		Validation Set II (*n* = 251)	
		HR (95% CI)	*p* Value	HR (95% CI)	*p* Value	HR (95% CI)	*p* Value
Gender	Female Male	1.823 (1.305–3.081)	0.819	1.059 (0.456–2.474)	0.782	1.808 (0.817–2.88)	0.502
Age	≤65 65 < x < 70 ≥70	2.267 (1.583–4.111)	0.232	2.262 (1.954–4.453)	0.291	2.132 (1.447–3.302)	0.122
Binet stage	A B C	2.119 (1.934–4.482)	0.071	2.829 (1.287–6.015)	<0.01	3.492 (2.338–5.963)	<0.01

CI: Confidence Intervals, CircRNA: Circular RNA, CLL: Chronic Lymphocytic Leukemia, and HR: Hazard Ratio.

**Table 5:** Univariate and Multivariate Cox regression analysis of overall survival of circRNAs signature as individual biomarkers and in the form of panels in CLL patients.

	Training Set (*n* = 100)				Validation Set I (*n* = 180)				Validation Set II (*n* = 203)			
	Unadjusted		Adjusted		Unadjusted		Adjusted		Unadjusted		Adjusted	
	HR (95% CI)	*p* Value	HR (95% CI)	*p* Value	HR (95% CI)	*p* Value	HR (95% CI)	*p* Value	HR (95% CI)	*p* Value	HR (95% CI)	*p* Value
CircKAT6A High vs. Low	3.156 (2.225–4.306)	<0.01	2.374 (1.996–3.852)	0.025	3.711 (3.205–4.928)	<0.01	3.356 (2.341–4.070)	<0.01	4.816 (3.611–6.048)	<0.001	4.525 (3.091–5.121)	<0.001
CircLNPEP High vs. Low	4.113 (2.346–5.288)	<0.001	3.222 (2.271–3.578)	<0.01	4.869 (3.559–5.251)	<0.001	3.848 (3.192–4.645)	<0.01	5.138 (4.592–6.09)	<0.001	4.279 (3.956–4.739)	<0.001
CircMDM2 High vs. Low	2.309 (1.884–4.093)	0.033	1.937 (1.509–3.583)	0.048	3.919 (2.704–5.191)	<0.01	2.974 (2.117–4.632)	<0.01	4.257 (3.816–5.534)	<0.001	3.746 (3.164–4.816)	<0.01
HMGCS2 High vs. Low	2.124 (1.394–4.922)	0.039	2.053 (1.197 –3.872)	0.042	3.983 (2.888–5.307)	<0.001	3.136 (2.255–4.333)	<0.01	4.249 (3.483–5.525)	<0.001	3.958 (3.417–4.783)	<0.001
CircMYH9 High vs. Low	3.259 (1.847–4.303)	<0.01	2.775 (1.362–3.983)	0.011	4.202 (2.581–5.236)	<0.001	3.371 (2.016–4.176)	<0.01	4.794 (3.503–5.596)	<0.001	4.015 (3.139–5.131)	<0.001
Binet Risk High vs. Low	1.317 (1.052–2.338)	0.09			2.933 (2.350–4.175)	<0.01			3.686 (2.802–4.974)	<0.01		
Panel High vs. Low	6.083 (5.152–8.023)	<0.001	4.193 (3.122–5.243)	<0.001	7. 517 (6.117–8.886)	<0.001	5.432 (4.716–6.915)	<0.001	8.294 (7.503–9.167)	<0.001	6.992 (5.9365–8.049)	<0.001

CI: Confidence Intervals, CircRNA: Circular RNA, CLL: Chronic Lymphocytic Leukemia, and HR: Hazard Ratio.

## Data Availability

The data presented in this study are openly available in Gene Expression Omnibus (GEO) (URL: https://www.ncbi.nlm.nih.gov/geo/, accessed on 7 September 2022).

## Supplementary Materials

The following supporting information can be downloaded at: https://www.mdpi.com/article/10.3390/cancers15051554/s1, Table S1: Differentially expressed RNAs; Table S2: GO Biological Process; Table S3: WikiPathway Annotations; Table S4: Drug Sensitivity Prediction.
